# A network-based method to evaluate quality of reproducibility of differential expression in cancer genomics studies

**DOI:** 10.18632/oncotarget.5987

**Published:** 2015-11-09

**Authors:** Robin Li, Xiao Lin, Haijiang Geng, Zhihui Li, Jiabing Li, Tao Lu, Fangrong Yan

**Affiliations:** ^1^ Research Center of Biostatistics and Computational Pharmacy, China Pharmaceutical University, Nanjing, Jiangsu, China; ^2^ State Key Laboratory of Natural Medicines, China Pharmaceutical University, Nanjing, Jiangsu, China

**Keywords:** cancer genomics, gene expression, overlapping genes, pagerank, reproducibility

## Abstract

**BACKGROUND:**

Personalized cancer treatments depend on the determination of a patient's genetic status according to known genetic profiles for which targeted treatments exist. Such genetic profiles must be scientifically validated before they is applied to general patient population. Reproducibility of findings that support such genetic profiles is a fundamental challenge in validation studies. The percentage of overlapping genes (POG) criterion and derivative methods produce unstable and misleading results. Furthermore, in a complex disease, comparisons between different tumor subtypes can produce high POG scores that do not capture the consistencies in the functions.

**RESULTS:**

We focused on the quality rather than the quantity of the overlapping genes. We defined the rank value of each gene according to importance or quality by PageRank on basis of a particular topological structure. Then, we used the *p*-value of the rank-sum of the overlapping genes (PRSOG) to evaluate the quality of reproducibility. Though the POG scores were low in different studies of the same disease, the PRSOG was statistically significant, which suggests that sets of differentially expressed genes might be highly reproducible.

**CONCLUSIONS:**

Evaluations of eight datasets from breast cancer, lung cancer and four other disorders indicate that quality-based PRSOG method performs better than a quantity-based method. Our analysis of the components of the sets of overlapping genes supports the utility of the PRSOG method.

## INTRODUCTION

Personalized cancer treatment decisions rely on the identification of gene mutations that drive tumorigenesis in a given patient and are based on comparisons of driver gene mutations across populations of patients [1]. For example, in non-small cell lung cancer, the efficacy of a *BRAF* mutant allele selective inhibitor is related to the *BRAF* V600E mutational status in the cancer cells [2]. Other research has shown that it is important to determine the status of both *BRAF* and *RAS* before using RAF inhibitors [3–5]. Determining the status of genes to inform cancer treatment decisions yet faces many challenges [6]. Some studies have shown that low frequency mutations, which are not found in every patient, can act as drivers of disease. In breast cancer, for example, the hotspot *AKT*1 E17K mutation occurs in only about 3% of primary breast cancers; however, that gene is an important part of the PI3-kinase-AKT-mTOR pathway, which is frequently mutated in breast cancer [7].

Nussinov et al. illustrated that actionable mutations should include not only those detected as drivers of disease, but also some presumed to be passengers, which may actually be ‘latent driver’ mutations that are additively pathogenic under some conditions [8]. They advocate the analysis of mutations within the structural architecture of molecular pathways. Identifying such ‘latent driver’ mutations can inspire the development of more personalized treatments.

Like compartmental model in pharmacokinetics, a stable model, which produces consistent results from different studies, provides a foundation for the translation of gene expression data into clinical practice. The performance of such confirmatory studies and the transition to clinical practice require that microarray data from different laboratories are comparable and reproducible [9]. However, the sets of differentially expressed genes (DEGs) obtained from studies in the same disease have differed widely and have often had only a few genes in common [10–14]. This frustrating phenomenon has raised doubts about the reliability and robustness of the predictive gene lists reported from studies of microarray data [15]. The MicroArray Quality Control (MAQC) project was initiated to address these concerns, as well as other performance and data analysis issues [12]. This study has provided valuable information but is yet far from comprehensive [16].

Both the concepts and metrics used to determine the reproducibility of DEGs are not uniformly defined [11, 12, 17]. Most metrics for evaluating the reproducibility between two DEG lists, such as the percentage of overlapping genes (POG), depend on the quantity of overlapping and related genes. The potential assumption underlying these metrics is that genes in both lists have the same position of importance in the development of a disease. However, a gene may play different roles in various diseases and even show inconsistent function in different stages of a single disease. For example, the Bcl-2 family includes key regulators of apoptosis, both antiapoptotic and proapoptotic genes [18, 19]. Chen et al. illustrated that POG does not reflect the accuracy of a selected DEG list [20]. A gene that is not identified as differentially expressed in two studies (a non-overlapping gene) may truly be differentially expressed, and an overlapping gene may actually not be differentially expressed, depending on the cutoff for the number of genes selected. POG is limited as a selection criterion because of its dependence on the size of the set of DEGs. Thus, a POG score calculated under such conditions will produce misleading results, such as misdiagnose of breast cancer patient by comparing genes expression with diagnosed breast cancer patients, and possibly lead to ineffective treatments.

In this paper, we evaluate the quality rather than the quantity of overlapping genes when comparing two or more sets of DEGs. We define the rank value of each gene as importance or ‘quality’ by PageRank on basis of a particular topological structure. We propose the *p*-value of the rank-sum of the overlapping genes (PRSOG) method to evaluate the reproducibility of DEGs. We analyze the components of the set of overlapping genes, including whether a gene is significant, common, or incorrectly listed (a wrong gene), to increase the reliability of the PRSOG method.

## RESULTS

### Reproducibility of studies for eight datasets

Using eight datasets from two platforms, we analyzed twelve experiments. The eight datasets are available at Gene Expression Omnibus (GEO) [21] and are described in detail under Materials and Methods. To ensure the comparability of PRSOG and POG between experiments, we used the significance analysis of microarrays (SAM) method [22] to identify the list of DEGs for each dataset, using fewer than 1000 DEGs. The false discovery rates [23] in all datasets were less than 1%, with the exception of an 8.2% false discovery rate in dataset GSE28686 (from a study of the illicit use of methcathinone) [24].

#### Experiment 1.1: breast cancer

In this experiment, we detected 963 and 856 DEGs in the respective datasets GSE36295 [25] and GSE39004 [26]. We measured the POG in two directions. For the list of DEGs detected in the first dataset, GSE36295, we measured the percentage of genes that also appeared in the second dataset, GSE39004, and called that score POG_12_. For the list of DEGs detected in the second dataset, GSE39004, we measured the percentage of genes that also appeared in the first dataset, GSE36295, and called that score POG_21_. The respective POG_12_ and POG_21_ scores were 0.32 and 0.36 in experiment 1.1.

#### Experiment 2.1: lung cancer, with different tumor subtypes

In this experiment, we detected 916 and 933 DEGs in respective datasets GSE18842 [27] and GSE19804 [28]. The corresponding POG_12_ and POG_21_ scores were 0.39 and 0.38.

#### Comparative experiments

Using SAM, we detected 859, 836, 834 and 910 DEGs, respectively, in datasets GSE25041 (study of adipose tissue) [29], GSE28686 (study of illicit methcathinone use) [24], GSE30999 (study of psoriasis) [30] and GSE19743 (study of burn injuries) [31].

The POG scores for all 12 experiments are listed in Table 1, where we observe low POG scores in every experiment, which suggests that most of the genes identified as being differentially expressed were inconsistent when comparing the first dataset to the second dataset. That finding of low reproducibility in microarray analyses has been observed in many studies [14, 32]. Furthermore, we found differences in the POG scores from experiments in the same disease and in different diseases. The POG scores in experiments 1.1 and 2.1, which evaluated two datasets for the same disease (breast cancer and lung cancer, respectively), were greater than 0.3; however, most of the other POG scores from different diseases were less than 0.1.

**Table 1:** POG scores of 12 experiments using 8 datasets

Platform: GPL6244				Platform: GPL570			
Experiment	GEO accession	*POG_12_	**POG_21_	Experiment	GEO accession	*POG_12_	**POG_21_
Experiment 1.1	1. GSE36295 2. GSE39004	0.32	0.36	Experiment 2.1	1. GSE18842 2. GSE19804	0.39	0.38
Experiment 1.2	1. GSE25401 2. GSE28686	0.08	0.09	Experiment 2.2	1. GSE30999 2. GSE19743	0.06	0.06
Experiment 1.3	1. GSE25401 2. GSE36295	0.09	0.08	Experiment 2.3	1. GSE18842 2. GSE30999	0.06	0.07
Experiment 1.4	1. GSE25401 2. GSE39004	0.10	0.10	Experiment 2.4	1. GSE19804 2. GSE30999	0.04	0.04
Experiment 1.5	1. GSE28686 2. GSE36295	0.06	0.05	Experiment 2.5	1. GSE18842 2. GSE19743	0.12	0.12
Experiment 1.6	1. GSE28686 2. GSE39004	0.06	0.06	Experiment 2.6	1. GSE19804 2. GSE19743	0.04	0.04

* POG_12_ score represents the reproducibility of a DEG list detected in dataset 2 when evaluating it in dataset 1

** POG_21_ score represents the reproducibility of a DEG list detected in dataset 1 when evaluating it in dataset 2

Using the POG score can lead to false discoveries in clinical datasets. In experiment 2.1 for non-small cell lung cancer, we obtained POG scores of 0.39 and 0.38, which were the highest POG scores in all the experiments. These results suggest that the reproducibility of DEGs from lung cancer datasets was the best of all the experiments. However, the tumor subtypes represented in the two datasets in experiment 2.1 were different. Dataset GSE18842 consisted of 69.6% squamous cell carcinomas in a total of 46 tumor samples and dataset GSE19804 consisted of 93.3% adenocarcinomas in 60 tumor samples. Many studies have demonstrated differences between the squamous cell carcinoma subtype and the adenocarcinoma subtype of non-small cell lung cancer, especially on the molecular level [33–36].

### Quality of reproducibility of DEG lists

There are apparent drawbacks to analyze reproducibility basing on the number of overlapping genes in DEG lists. Genes have different roles and functions in diseases and these functions should be discriminated in the evaluation of reproducibility. A gene's rank, calculated by PageRank on basis of a particular network or topological structure, represents a single gene's importance or quality.

The total ranking value in the topological structure of an experiment is 1, and a single gene in a network with *N* genes has a rank of 1/*N* before running PageRank. After iteration in the network built according to correlation coefficients larger than 0.7, for example, the values of the rank-sum of the overlapping genes (RSOG) for experiment 1.1 and experiment 2.1 are respectively 0.26 and 0.23. The RSOG indicates the importance of *k* overlapping genes in the total rank. Because of the different topological structures and dependence on the number of genes in the network, the RSOG values are useless for strictly evaluating the quality of reproducibility.

We ran simulations 10,000 times to pick *k* genes in the rank pool and built the distribution of the RSOG of *k* genes in an experiment. The central limit theorem ensured a normal distribution of the RSOG, which is shown in Supplementary Figure S1. We then obtained the PRSOG.

The PRSOGs of experiments 1.1 are 1.11 × 10^−16^, converging to 0 and 2.1 are 0.88. These results indicate that experiment 1.1 in breast cancer had successful reproducibility, with a significant PRSOG less than 0.01. In contrast, experiment 2.1, which evaluated different subtypes of non-small cell lung cancer, had low reproducibility. Another explanation of the PRSOG is that, in experiment 1.1, the 0.26 RSOG was not randomized and had statistical significance to cover the most important genes in breast cancer; whereas the 0.23 RSOG in experiment 2.1 occurred randomly and thus it was hard to achieve successful reproducibility. In Table 2, we list the mean POG, RSOG, distribution of RSOG, and PRSOG in all 12 experiments when the correlation coefficient was 0.7. Compared with the 10 experiments for different disorders, experiment 2.1 had the highest POG score, which meant that it had the highest number of overlapping genes among those 11 experiments. However, all of the experiments had statistically insignificant PRSOGs, which indicates unsuccessful reproducibility. Such results suggest that the PRSOG method has a strict threshold for judging the success of reproducibility.

**Table 2:** *P*-value of rank-sum of overlapping genes (PRSOG) of all 12 experiments when correlation coefficient is 0.7

Experiment	Mean POG	RSOG	RSOG Distribution		PRSOG	Experiment	Mean POG	RSOG	RSOG Distribution		PRSOG
			Mean	SD					Mean	SD	
1.1	0.34	0.26	0.21	0.0070	1.11 × 10^−16^	2.1	0.38	0.23	0.24	0.0067	0.88
1.2	0.085	0.047	0.046	0.0009	0.19	2.2	0.060	0.030	0.033	0.0022	0.84
1.3	0.085	0.045	0.046	0.0005	0.94	2.3	0.065	0.031	0.034	0.0024	0.83
1.4	0.100	0.051	0.053	0.0004	0.99	2.4	0.040	0.019	0.019	0.0020	0.58
1.5	0.055	0.030	0.029	0.0027	0.47	2.5	0.120	0.053	0.063	0.0035	0.99
1.6	0.060	0.027	0.030	0.0031	0.81	2.6	0.040	0.015	0.019	0.0020	0.95

We also built the gene network using correlation coefficients of 0.5, 0.6, 0.8 and 0.9. The PRSOGs obtained when using these different correlation coefficients in experiments 1.1 and 2.1 are shown in Table 3. The PRSOGs for experiment 1.1 were statistically significant and those for experiment 2.1 were not statistically significant when using these correlation coefficients. When we used the correlation coefficient 0.6, we obtained the best PRSOG in each experiment, meaning the smallest value, the most statistical significance, or the highest reproducibility.

**Table 3:** *P*-value of rank-sum of overlapping genes (PRSOG) of experiments 1.1 and 2.1, with different correlation coefficients

Experiment 1.1: breast cancer						Experiment 2.1: different subtypes of non-small cell lung cancer					
Correlation Coefficient	Mean POG	RSOG	RSOG Distribution		PRSOG	Correlation Coefficient	Mean POG	RSOG	RSOG Distribution		PRSOG
			Mean	SD					Mean	SD	
0.5	0.34	0.24	0.21	0.0046	2.22 × 10^−16^	0.5	0.38	0.24	0.24	0.0043	0.81
0.6	0.34	0.26	0.21	0.0056	0	0.6	0.38	0.24	0.24	0.0053	0.79
0.7	0.34	0.26	0.21	0.0070	1.11 × 10^−16^	0.7	0.38	0.23	0.24	0.0067	0.88
0.8	0.34	0.28	0.21	0.0097	3.32 × 10^−13^	0.8	0.38	0.22	0.24	0.0093	0.95
0.9	0.34	0.32	0.21	0.0271	1.05 × 10^−5^	0.9	0.38	0.18	0.24	0.0268	0.98

**Figure 1:**
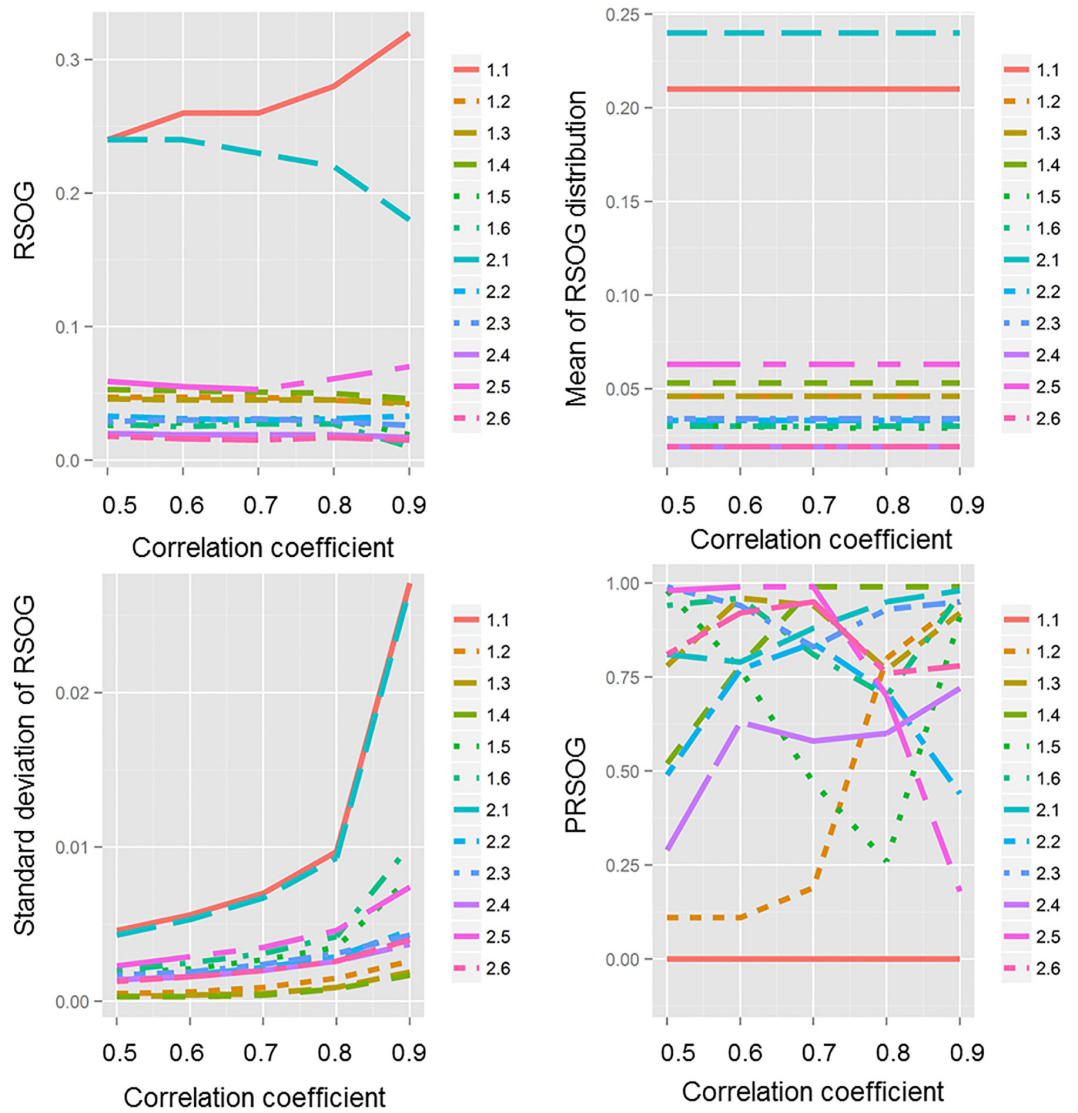
The effect of correlation coefficient on RSOG, mean of RSOG distribution, standard deviation of RSOG distribution and PRSOG The *x*-axis is the correlation coefficient from 0.5 to 0.9 by increments of 0.1; the y-axis is either the RSOG, mean of RSOG distribution, standard deviation of RSOG distribution or PRSOG of the 12 experiments.

To find the effect of the correlation coefficient on the PRSOG method, we plotted the RSOG, mean of the RSOG distribution, standard deviation of the RSOG distribution and PRSOG values (Figure 1). We found that the mean of the RSOG distribution was stable when the network was defined, but the standard deviation of the RSOG distribution increased with a larger correlation coefficient, which meant more edges in the network and larger differences between the ranks of two genes. This result indicates that the PRSOG is influenced by the RSOG and the standard deviation of the RSOG, which are both determined by the underlying topological structure. The inference suggests that a good fit of the network to the underlying biological process is helpful for evaluating the reproducibility of microarray studies. We found the same results in another 10 experiments (Supplementary Tables S1–S10).

### Analyzing the importance of overlapping genes

We wanted to determine the reason behind completely different PRSOG values in the breast cancer data compared to the lung cancer data when both diseases had similar POG scores, indicating similar numbers of overlapping genes. Therefore, we analyzed the importance of every gene among the set of overlapping genes.

**Figure 2:**
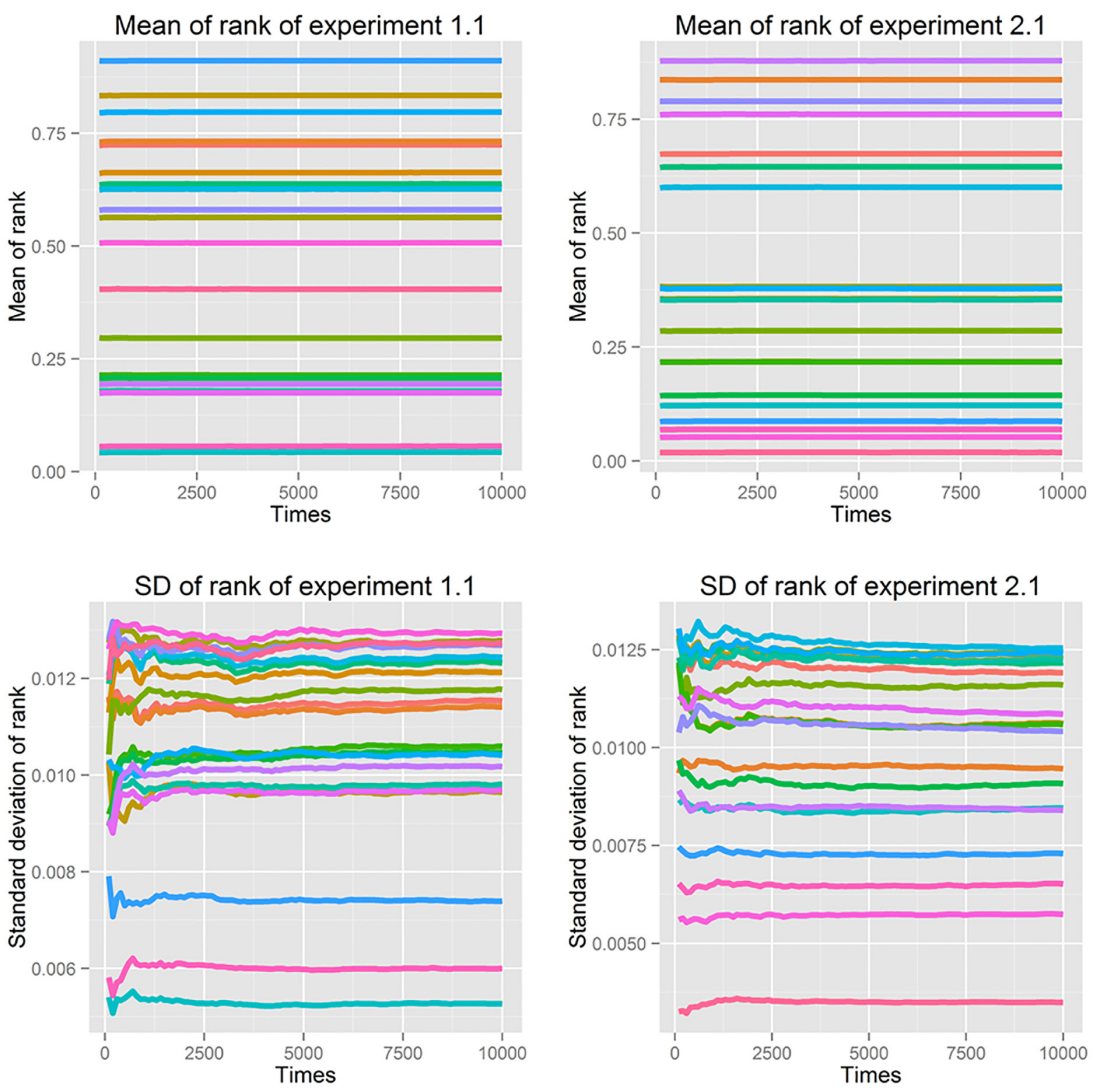
Mean and standard deviation of *p*-value in 10,000 resampling procedures of 20 genes selected randomly from experiments 1.1 and 2.1, using correlation coefficient 0.7

First, we fit power-law, log-normal, and exponential distributions of the genes' ranks in the gene pool of the network. Table 4 lists the results when the correlation coefficient was 0.7. It is clear that the power-law distribution and the exponential distribution are good fits for Kolmogorov-Smirnov test *p*-values larger than 0.05. The log-normal distribution had a statistical significance of 0.05 for just half of the experiments. When the correlation coefficient was 0.9, because of isolated genes that had no links, some genes with rank 0 (not exactly 0 but infinitely close to 0) made it impossible to fit the three distributions. The fitting results with correlation coefficients 0.5, 0.6, 0.8 and 0.9 are listed in Supplementary Tables S11–S14. Second, we used resampling procedure to calculate the *p*-value of a single gene, which represented the importance of that single gene (see Analysis and Classification of Overlapping Genes under Materials and Methods). We defined the significant genes, common genes and wrong genes using these *p*-values. We resampled the data 10,000 times and observed that the mean and standard deviation of the *p*-value converged to a stable value. The convergent processes of 20 genes selected randomly from experiments 1.1 and 2.1 are shown in Figure 2.

**Table 4:** Results of fitting power-law, log-normal, and exponential distributions with correlation coefficient 0.7

Experiment	Power-law distribution			Log-normal distribution				Exponential distribution		
	X_min_	Parameter	K-S	X_min_	Parameter 1	Parameter 2	K-S	X_min_	Parameter	KS
1.1	0.00141	65.0	0.067	0.00134	−6.587	0.0247	0.037*	0.00144	0.000742	0.667
1.2	0.00066	192.0	0.054	0.00066	−7.320	0.0083	0.032*	0.00069	0.000625	0.667
1.3	0.00059	526.0	0.093	0.00058	−7.439	0.0051	0.084	0.00061	0.000577	0.667
1.4	0.00062	620.0	0.122	0.00062	−7.381	0.0041	0.101	0.00063	0.000615	0.667
1.5	0.00115	99.9	0.086	0.00093	−6.870	0.0700	0.053	0.00118	0.000648	0.667
1.6	0.00141	65.0	0.067	0.00134	−6.587	0.0247	0.037*	0.00144	0.000742	0.667
2.1	0.00141	19.0	0.082	0.00125	−6.631	0.0962	0.029*	0.00162	0.000693	0.667
2.2	0.00099	53.4	0.075	0.00097	−6.922	0.0268	0.029*	0.00105	0.000619	0.667
2.3	0.00089	177.0	0.056	0.00087	−7.033	0.0117	0.048*	0.00092	0.000683	0.667
2.4	0.00095	141.0	0.062	0.00094	−6.963	0.0114	0.041	0.00098	0.000664	0.667
2.5	0.00121	36.2	0.089	0.00113	−6.750	0.0500	0.038	0.00129	0.000631	0.667
2.6	0.00117	47.5	0.065	0.00097	−6.859	0.0726	0.0009**	0.00123	0.000607	0.667

K-S = *p*-value of Kolmogorov-Smirnov test, which is commonly used to compare a sample with a reference probability distribution or two samples;

* *p*-value of K-S test has statistical significance of 0.05;

** *p*-value of K-S test has statistical significance of 0.01.

**Figure 3:**
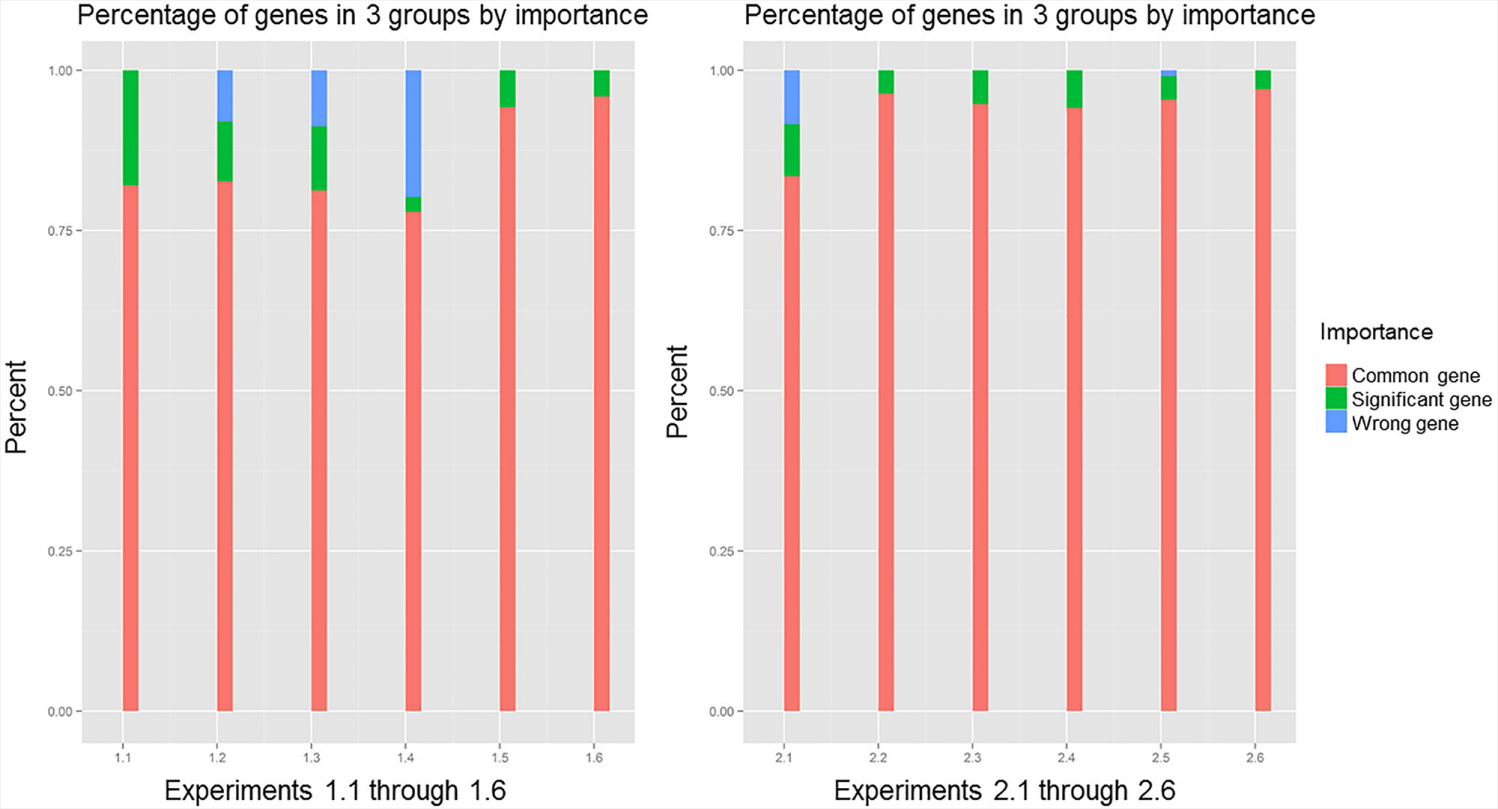
Components of overlapping genes in 12 experiments, with correlation coefficient 0.7; comparing experiments 1.1 and 2.1

Using the *p*-value calculated by resampling procedure, we classified the genes in the gene pool of the network into three classes with the following ranges of rank values: (0, 0.1], (0.1, 0.9] and (0.9, 1). The percentages of these three components in the 12 experiments when the correlation coefficient was 0.7 are plotted in Figure 3. In all 12 experiments, common genes occupied more than 75% of the overlapping genes that belonged to the gene pool, which contained 80% common genes according to the definition of the three kinds of genes we assessed. Increasing the percentage of significant genes is the key to improving the PRSOG; whereas increasing the percentage of wrong genes reduces the PRSOG. Thus, for successful reproducibility of a microarray study, the network gene pool should include more significant genes and few wrong genes. Comparing experiments 1.1 and 2.1, we found similar percentages of common genes; however, the visible difference was the proportion of wrong genes. Experiment 1.1 had almost no wrong genes, but experiment 2.1 had the same percentages of significant genes and wrong genes. It was thus easy to comprehend the cause of the outcome observed in experiment 2.1, which analyzed different tumor subtypes in lung cancer. The same conclusions were obtained when we used correlation coefficients 0.5, 0.6, 0.8 and 0.9 (Supplementary Figures S2–S5).

The results of our analyses of 8 datasets in 6 diseases/disorders show that diseases in different subtypes with relatively high POG scores had statistically insignificant PRSOGs, which indicated low reproducibility. In addition, the effect of the correlation coefficient on network building suggests that a better approximation of the network underlying the biological process is helpful when evaluating reproducibility.

## DISCUSSION

The cancer treatment depends on a patient's cancer mutation spectrum and the comparison of that spectrum with known statistical trends across the relevant population of patients [37]. Theoretically, a high POG score is expected when comparing the results of two studies in the same cancer. When evaluating reproducibility, a major shortcoming of the POG criterion is that it treats all significant genes the same and simply counts them. We compared the DEG lists from different studies in the same disease by assigning each gene the rank that represents its contribution to the disease. Then, we used PRSOG method to evaluate the reproducibility of DEG lists between different studies.

The POG scores from 12 experiments showed a small number of genes that overlapped between two studies, which was consistent with the findings of former studies. Greater than 30% of the POG scores for the experiments in breast cancer and lung cancer were not high enough to apply in clinical practice. In other words, we could not ascertain whether the same results were achieved in the two studies. Using PRSOG method, we found that the quality of reproducibility between the two studies in breast cancer was high (a success), but that the quality of reproducibility between the two studies in different subtypes of lung cancer was low (a failure). Inconsistent findings in squamous cell carcinomas compared to adenocarcinomas of the lung have been investigated in omic studies [33–36]. Moreover, the diagnoses and treatments provided to patients with squamous cell carcinomas of the lung compared to adenocarcinomas of the lung are different [38, 39]. Thus, we suggest that the quality instead of the quantity of overlapping genes should receive more attention when evaluating reproducibility. Then, we studied the effect of the correlation coefficient, which could be treated as prior information of the disease, on the results. In a particular disease, a rational correlation coefficient, which could be replaced by a real gene network in a bioinformatics database, was helpful in achieving a better result. The correlation coefficient exerts varying influence on the *p*-value of the PRSOG method in different experiments, which indicates that the essential factor in this method is the specific type of disease. To determine why different judgments were made by PRSOG when similar numbers of overlapping genes were found in the experiments in breast cancer and lung cancer, we classified the genes into significant genes, common genes and wrong genes according to the rank values. The results indicate that both the presence of few wrong genes and few common genes in the overlapping genes is important to achieve a high level of reproducibility.

To our knowledge from relative works, there are two mainly methods of assessing the reproducibility of gene expression. The first method is POG method and derivative methods. In this paper, we compared PRSOG with POG and pointed out the principle problems of POG which had been discussed distinctly above. The second method is correlation coefficient method, simply such as Pearson Correlation, and derivative methods. However, the correlation coefficient method has the totally different prerequisite from PRSOG and POG. In Pearson Correlation, correlative correlation between gene list 1 and gene list 2 was calculated to assess the reproducibility. This calculating procedure needed that the two lists had the same elements that, in POG's opinion, meant the POG score between the two lists was 1. However, the basic assumption in our paper was that the POG between two gene lists was small. Above all, it is meaningless to compare PRSOG with the second method. There are many ways to infer or ‘reverse-engineer’ a gene network from expression profiles, such as using Bayesian networks [40, 41], information theory [42, 43], ordinary differential equations [44] and methods based on databases such as Gene Ontology [42, 45] and the Kyoto Encyclopedia of Genes and Genomes [46]. Further investigations are warranted to study the reproducibility of DEG lists produced through different approaches to network building. It has been suggested that using thousands of samples of a disease will generate a reproducible DEG list [11]; however, such a list is hardly reproducible in small samples. The emergence of big data [47, 48], particularly in medicine and biology [49, 50], has improved data accessibility through the rapid generation of huge volumes and variety of omic data. Greater meaning in clinical applications, however, will require a powerful method to evaluate reproducibility in small samples, especially in personalized treatments for which little biopsy tissue is available for producing huge datasets.

## MATERIALS AND METHODS

### Datasets and selection of DEGs

To remove the needless factors affecting the results, we shrink the backgrounds of datasets as possible. There were three main factors of datasets: the platform, the preprocessing of chips and the method of DEGs selection. We selected the datasets, which used the same platform, into the same group. For preprocessing of chips, because the GEO used the same and standard process for the same platform, GEO was chosen as the only database to select datasets. In addition, in SAM, the method DEGs selection used in this paper, which was discussed below, because paired data and unpaired data had the different principles, datasets in the same group would be selected if they had the same data type. It should be noted that in such condition above the quantity of datasets was small. Above all, we obtained the eight datasets used in this paper from GEO [21]. The first group of four datasets represent two-class, unpaired data and the second group of four datasets represent paired data.

The first group of four datasets had been collected from three different medical disorders: breast cancer, obesity, and the illicit use of methcathinone. We used the datasets of human obesity and the illicit use of methcathinone to compare the metric of reproducibility with the breast cancer dataset. Two datasets, GSE36295 [25] and GSE39004 [26], described breast cancer, and the remaining two datasets, GSE25401 [29] and GSE28686 [24], provided information on human obesity and the illicit use of methcathinone, respectively. GSE25401 included biopsy data from 26 non-obese women and 30 obese women, in which the microRNA from adipose tissue was regarded as the regulator of the production of chemokine (C-C motif) ligand 2 (CCL2) in human obesity [29]. GSE28686 contained data from 20 methcathinone users and 20 matched controls, representing the study of the RNA expression profiles in peripheral blood samples to reveal the effect of methcathinone on the immune system [24]. For each medical disorder, we analyzed only data that were available from the same platform.

The second group of four datasets had been collected from three other medical disorders: non-small cell lung cancer, psoriasis, and severe burn injuries. The non-small cell lung cancer datasets were GSE18842 [27], characterized by 44 paired tumors and controls as well as three unpaired samples, and GSE19804 [28], characterized by 120 paired tumor and normal tissue samples. GSE18842 contained 69.6% squamous cell carcinomas and GSE19804 had 93.3% adenocarcinomas. The remaining two datasets consisted of samples of moderate-to-severe psoriasis, GSE30999 [30], and samples of severe burn injuries, GSE19743 [31].

The datasets we analyzed are summarized in Table 6. Because of non-available of missing value in network building described in below method section, we used the k-Nearest Neighbor (kNN) imputation algorithm (*k* = 15) to replace any missing data in the datasets [51].

**Table 5:** Definitions of power-law, exponential and log-normal distributions

Name	Distribution p(x)=Cf(x)	
	f(x)	C
Power-law	$x^{-\alpha }$	$(\alpha -1)x_{\min}^{\alpha -1}$
Exponential	$e^{-\lambda x}$	$\lambda e^{-\lambda x_{\min}}$
Log-normal	$\frac{1}{x}\exp[-\frac{{\left(lnx-\mu \right)}^{2}}{2\sigma^{2}}]$	$\sqrt{\frac{2}{\pi \sigma^{2}}}[\mathrm{erfc}(\frac{\ln x_{\min}-\mu }{\sqrt{2}\sigma })]$

**Table 6:** The summarized information of datasets from GEO

GEO ID	Disease	Tissue	Samples Size	Platform ID
GSE36295	Breast cancer	Breast tissues	53	GPL6244
GSE39004	Breast cancer	Breast tissues	180	GPL6244
GSE25401	Human obesity	Adipose tissue	56	GPL6244
GSE28686	Illicit methcathinone	Blood tissue	40	GPL6244
GSE18842	Lung cancer	Lung tissue	91	GPL570
GSE19804	Lung cancer	Lung tissue	120	GPL570
GSE30999	Psoriasis	Skin biopsy	170	GPL570
GSE19743	Burn injury	Blood sample	177	GPL570

We used the SAM method (samr_2.0 R packages) [22] to select DEGs in each dataset. In the SAM method, users pick a fixed threshold and then identify the significant genes. Recently, many researches [52–54], which selected the differential expressed genes by SAM, showed that SAM was a popular and powerful method in expression studies. Distinct methods for selecting DEGs, such as the analysis of variance [55] and empirical Bayes with t-statistics [56, 57], may capture different statistical aspects of gene expression changes and contribute to the observed inconsistency between the derived DEGs [58, 59]. An analysis of the differences between these methods is beyond the scope of this paper; however, it would be a good topic for future research. It also should be noted that an advantage of SAM was the different strategies for paired data and unpaired data, whereas FC and *t*-test were not.

### PageRank and quality of reproducibility

**Figure 4:**
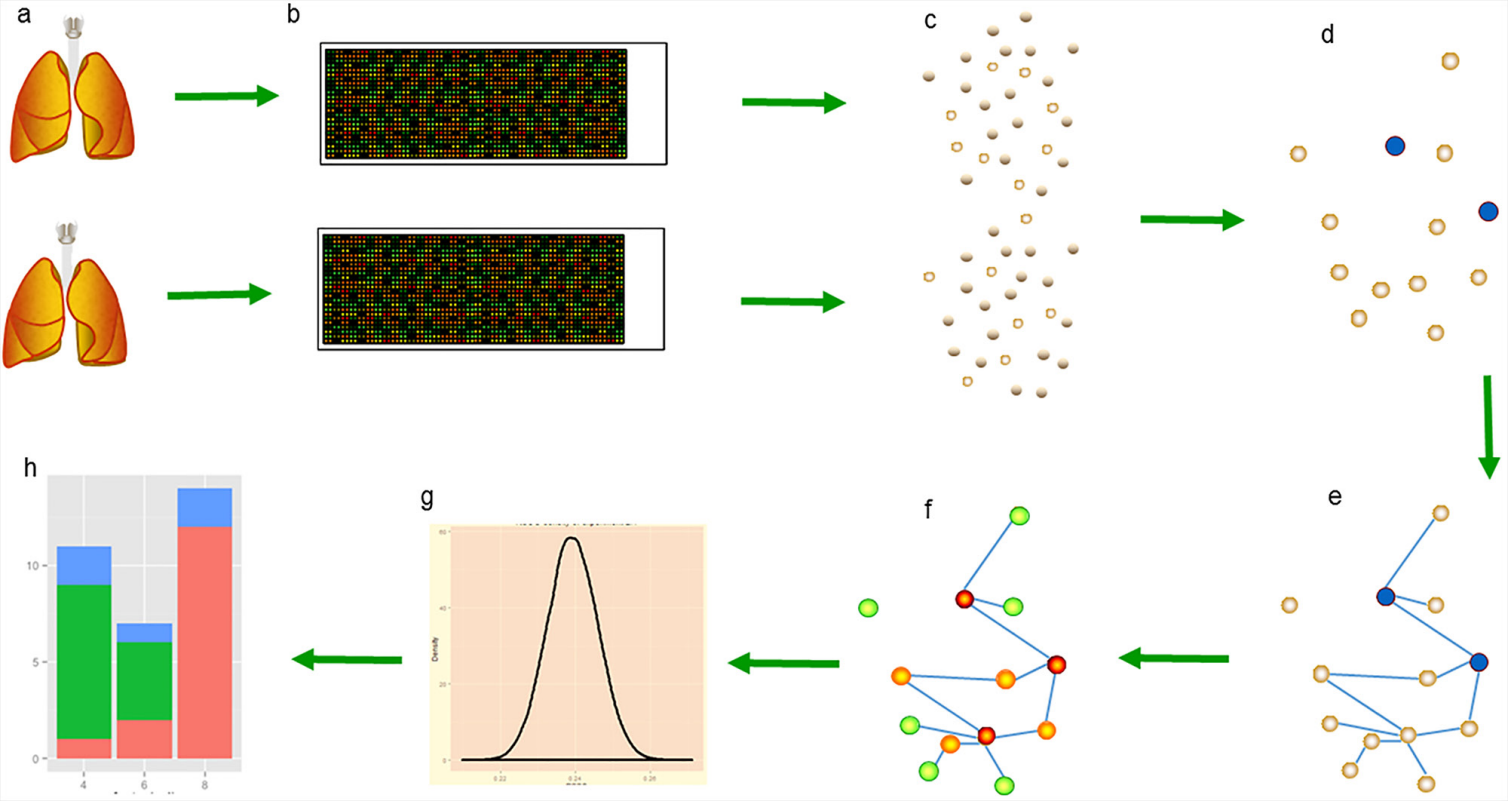
The PRSOG process in non-small cell lung cancer **a.** Two lung pictures represent two studies in non-small cell lung cancer by different labs; our experiment 2.1 assesses the reproducibility of these two studies. **b.** The RNA expression data of the two studies uses the same platform to ensure the same gene background. **c.** The significant genes (empty circles) in each dataset are calculated by SAM, controlling the quantity in 1000. **d.** Blue circles are reproducible genes among significant genes found in both studies. **e.** Building the network of this gene pool by correlation coefficient. **f.** Calculating the rank of every gene by PageRank; a warmer color indicates a more important role in the network. **g.** Assuming *k* overlapping genes in the gene pool, we resample *k* genes in the gene pool to build the distribution of *k* genes and then calculate the *p*-value of the rank sum of theses *k* overlapping genes. **h.** Classifying genes in the gene pool into three kinds by rank value.

Methods that use the POG score count the genes that overlap between two lists of DEGs to measure the reproducibility of microarray studies. Each gene in the set of overlapping genes is treated equally in the POG criterion. However, a gene may play different roles in different diseases and have inconsistent functions in different stages of a single disease. Furthermore, within the set of overlapping genes, this method includes some genes that we call wrong genes because they are not significant for the particular disease of interest. The process we used in the PRSOG method, which is a qualitative approach, is illustrated in Figure 4.

Our first experiment was a study of the reproducibility of a list of DEGs between two datasets. The gene pool of the network in the experiment was the union of the significant DEGs from the two datasets. We used PageRank to calculate the rank of a gene, the importance of the gene in the relevant network in which it functions. Before implementing PageRank, we used the correlation coefficient to build the necessary topological structure underlying the biology process. We calculated the correlation coefficient [60] for each gene pair in the gene pool and defined the edges of the network as pairs of genes with correlation *r*. To represent different kinds of networks such as a Bayesian network and a Gene Ontology network, we calculated five networks in an experiment with correlation *r* ranging from 0.5 to 0.9, with increments of 0.1. The correlation coefficient network was an undirected graph and an edge in the coefficient network was two-sided if a directed graph was necessary.

PageRank is an algorithm used by Google search engines to rank websites. According to Google, PageRank works by counting the number and quality of links to a page to determine a rough estimate of the importance of the website. The underlying assumption is that more important websites are likely to receive more links from other websites [61].

The original Brin and Page model for PageRank used the hyperlink structure of the web to build a Markov chain with a primitive transition probability matrix **P**. The irreducibility of the chain guarantees the existence of the long-run stationary vector π^T^, known as the PageRank vector. It is well known that the power method applied to a primitive matrix would converge to this stationary vector [62].

At the initial network state, each gene has the same rank *r* = 1/N, where *N* is the number of genes in the network. The PageRank algorithm calculates the *r_i_* of the *i* gene according to the correlation coefficient network topology structure by the following equation:

$$PageRank(g_{i})=\frac{1-d}{N}+d\sum_{p_{j}}\frac{PageRank(g_{j})}{L(g_{j})}$$ (1)

where *g_1_, g_2_,…,g_n_* are the *N* genes in the network, *L(g_j_)* is the number of links from gene *j*, and d ∈ (0, 1) is a fixed parameter. In this paper, we used the value *d* = 0.85, which appears to be what was proposed by Google [61, 62]. **R**, the PageRank vector of *N* genes in the network, is the eigenvector of the matrix and also the solution of the following equation:

$$R=\left[\begin{array}{c} (1-d)/N\\ (1-d)/N\\ \vdots \\ (1-d)/N \end{array}\right]+d\left[\begin{array}{cccc} \ell (g_{1},g_{1}) & \ell (g_{1},g_{2}) & \cdots & \ell (g_{1},g_{N})\\ \ell (g_{2},g_{1}) & \ddots & & \vdots \\ \vdots & & \ell (g_{i},g_{j}) & \vdots \\ \ell (g_{N},g_{1}) & \cdots & \cdots & \ell (g_{N},g_{N}) \end{array}\right]R$$ (2)

where the adjacency function ℓ(g_i_, g_j_) is 0 if gene *i* does not link to *j*, and normalized such that for each *j*:

$$\sum_{i=1}^{N}\ell (g_{i},g_{j})=1$$ (3)

In an experiment, suppose *k* overlapping genes are detected between list 1 with length *l_1_* and list 2 with length *l_2_*. Then the POG score from list 1 to list 2 is *POG_12_ = k/l_1_* and the score from list 2 to list1 is *POG_21_ = k/l_2_*. The POG criterion does not have a convincing standard threshold for detecting whether the reproducibility of the experiment was a success or not because of the independence of the number of DEGs (the DEG length).

In this paper, we propose the PRSOG method. The PageRank vector **R** of the network with *N* genes obtained by the PageRank algorithm and the rank-sum of the overlapping genes (RSOG) score of the experiment is the sum of the *PageRank(g_i_)* of these *k* overlapping genes:

$$RSOP=\sum_{i=1}^{k}PageRank(g_{i})$$ (4)

To calculate the PRSOG, we resampled *k* rank values in PageRank vector **R** 100,000 times and built the distribution of the RSOG to calculate the *p*-value of the RSOG of the overlapping genes. According to central limit theorem, a normal distribution can be built by 10,000 times resampling. Then we can get the PRSOG as follow:

$$PRSOG=p\left(RSOG|\mu ,\sigma \right)=\frac{1}{\sigma \sqrt{2\pi }}e^{-\frac{{\left(RSOG-\mu \right)}^{2}}{2\sigma^{2}}}$$ (5)

Where μ and σ are the mean and standard deviation of RSOG by resampling 10,000 times.

The PRSOG, compared to α = 0.01, indicates the statistical significance of the experiment's reproducibility. A PRSOG less than α illustrates successful reproducibility in that the genes that overlap between two lists of significant genes cover the most important genes in the network gene pool.

### Analysis and classification of overlapping genes

The presence of wrong genes in the set of overlapping genes leads to an unreliable evaluation of reproducibility in microarray studies [63]. A strategy to solve this problem is to increase the influence of important genes in the evaluation of reproducibility and decrease the influence of wrong genes in that evaluation. Thus, it is important to distinguish wrong genes from important genes in the DEG list. The PRSOG method assigns a rank value to each gene in the gene pool, which is the importance of that gene, and evaluates the quality of reproducibility of microarray studies by strict statistical significance. However, the single rank value of a gene depends on the number of genes in the network. Hence, it is difficult to use the RSOG to consistently evaluate the importance of a given gene.

In our method, we recognize the *i* gene in the gene pool according to the *p-value[rank(g) > rank(g_i_)]*, which indicates the probability that a gene's rank is greater than the rank of gene *i* in the network gene pool. Then we classify genes in the gene pool into three categories: significant genes, with *p-value[rank(g) > rank(g_i_)]* less than or equal to 0.1; wrong genes, with *p-value[rank(g) > rank(g_i_)]* greater than or equal to 0.9; and common genes, with *p-value[rank(g) > rank(g_i_)]* between 0.1 and 0.9. We use a reference probability distribution to calculate the *p-value[rank(g) > rank(g_i_)]*.

The distribution of a wide variety of physical, biological, and man-made phenomena approximately follow a power-law over a wide range of magnitudes [64]. In statistics, a power-law is a functional relationship between two quantities, where one quantity varies as a power of another. Power-law has a mathematic form like follow

$$f(x)=ax^{-k}$$ (6)

where x is the rank value of a gene and *f* (*x*) the corresponding *p*-value of this gene.

It has been suggested that the power-law distribution of PageRank in web graphs is observed when the typical damping factor used in practice is between 0.85 and 0.90 [65, 66]. Therefore, we tried to fit the power-law distribution, exponential distribution and log-normal distribution to rank the vector **R**. We list the definitions of the three distributions in Table 5.

Although these three distributions were a good fit for the rank vector, the real underlying distribution might be another distribution. Therefore, we used resampling procedure to calculate the *p-value[rank(g) > rank(g_i_)]*, with a smaller *p*-value indicating a more important gene.

In our experiment, we resampled a new rank vector **R**^'^ with replacement in the ‘population’. We then calculated the *p*-value in the new rank vector **R**^'^ as follows:

$$P-value(rank(g)>rank(g_{i}))=\frac{\#(rank(g)>rank(g_{i}))}{N}$$ (7)

We performed the resampling procedure 10,000 times to obtain the mean and standard deviation of the *p*-value. We used the mean value to indicate the *p-value[rank(g) > rank(g_i_)]*.

## SUPPLEMENTARY FIGURES AND TABLES


