# Supplementary material for: A network-based method to evaluate quality of reproducibility of differential expression in cancer genomics studies
Source: Oncotarget. 2015 Nov 9;6(42):44714–27. doi: 10.18632/oncotarget.5987 (PMC4792587; doi:10.18632/oncotarget.5987)
Supplement: Supplementary file 1 [file oncotarget-06-44714-s001.pdf]

SUPPLEMENTARY FIGURES AND TABLES

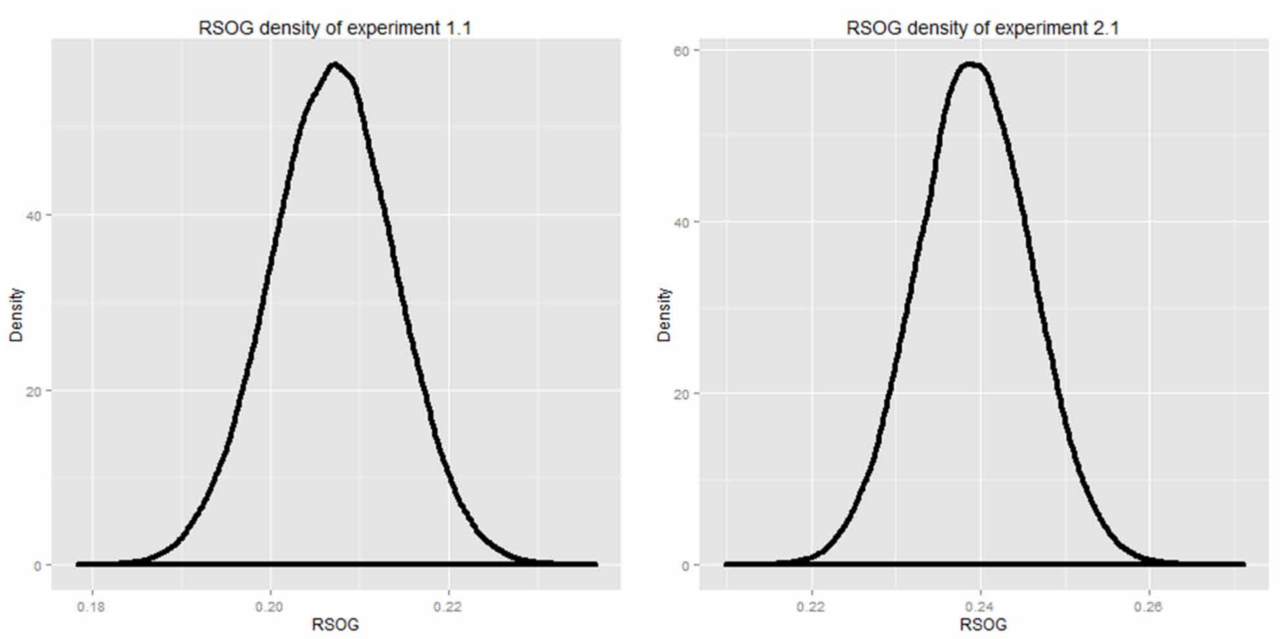

Supplementary Figure S1: The norm distribution of the RSOG of experiments 1.1 and 2.1 by running simulations 10,000 times to pick *k* genes in the rank pool.

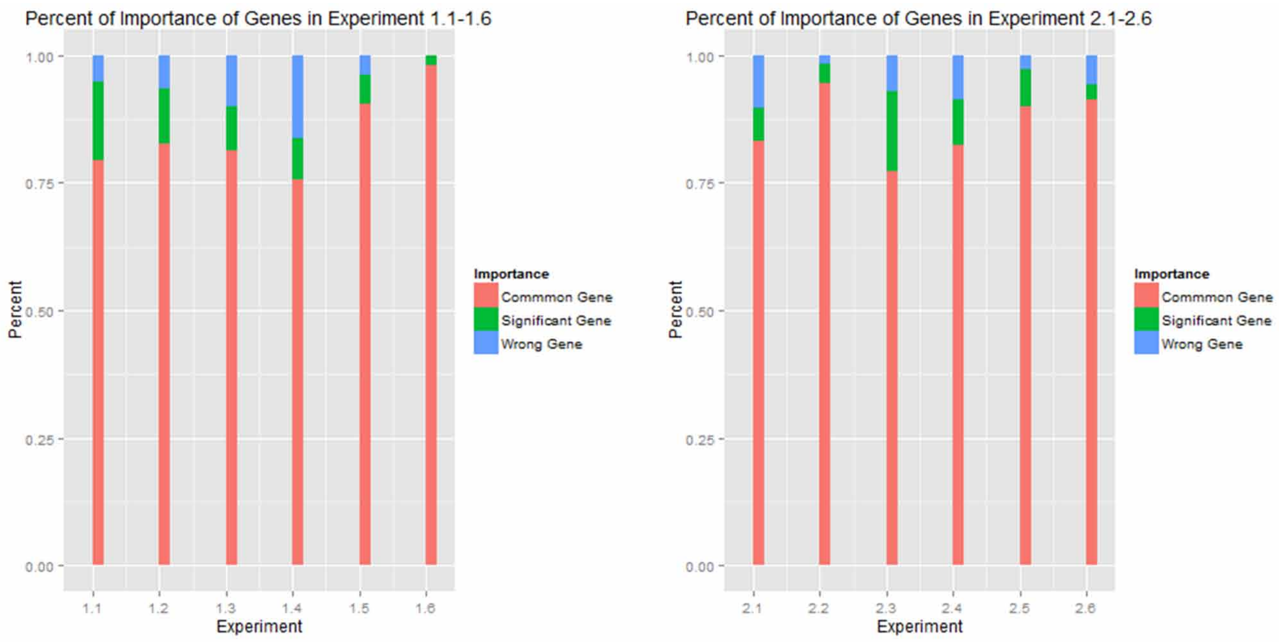

Supplementary Figure S2: Components of overlapping genes in 12 experiments, with correlation coefficient 0.5; comparing experiments 1.1 and 2.1.

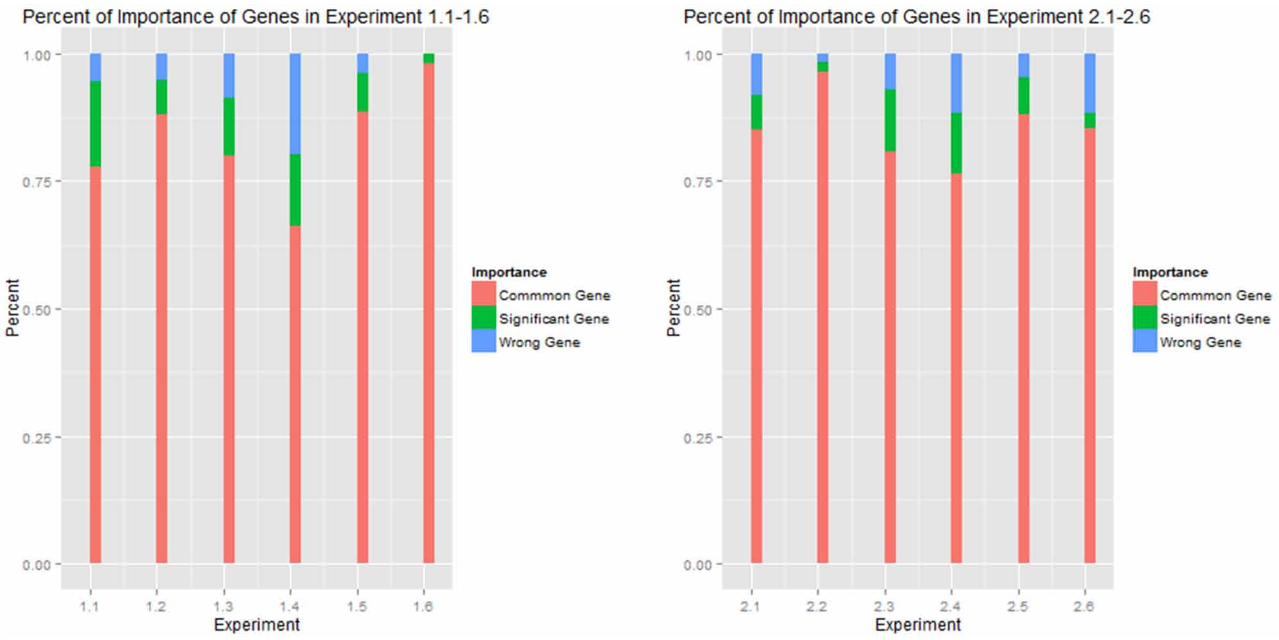

Supplementary Figure S3: Components of overlapping genes in 12 experiments, with correlation coefficient 0.6; comparing experiments 1.1 and 2.1.

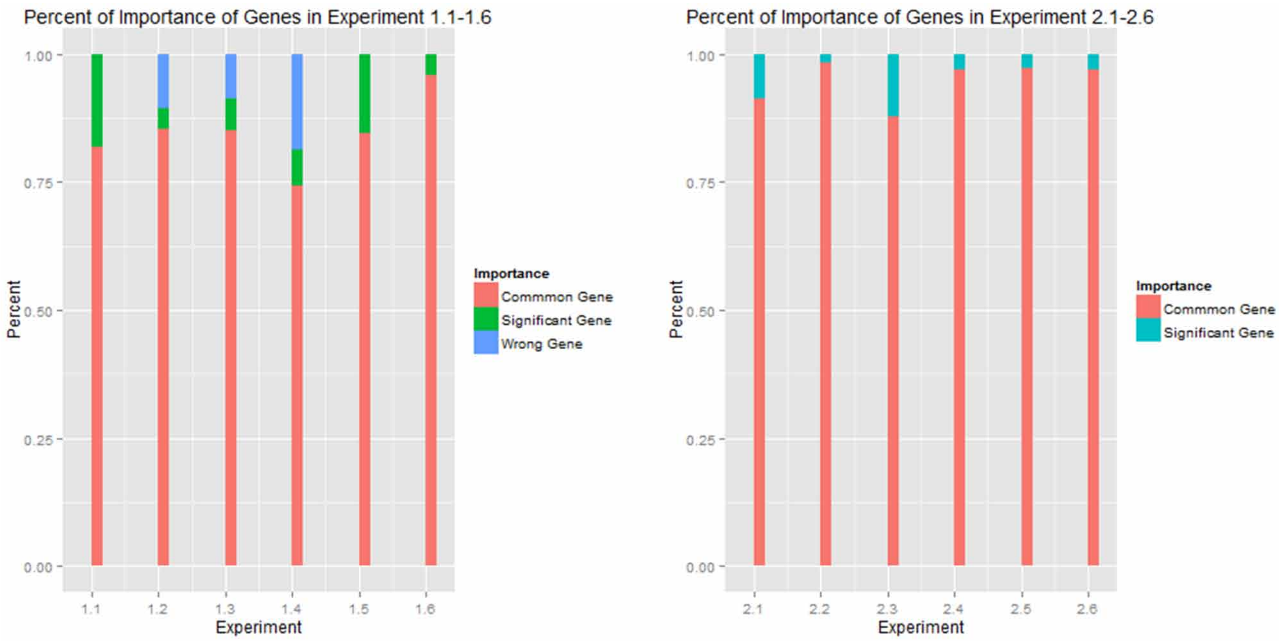

Supplementary Figure S4: Components of overlapping genes in 12 experiments, with correlation coefficient 0.8; comparing experiments 1.1 and 2.1.

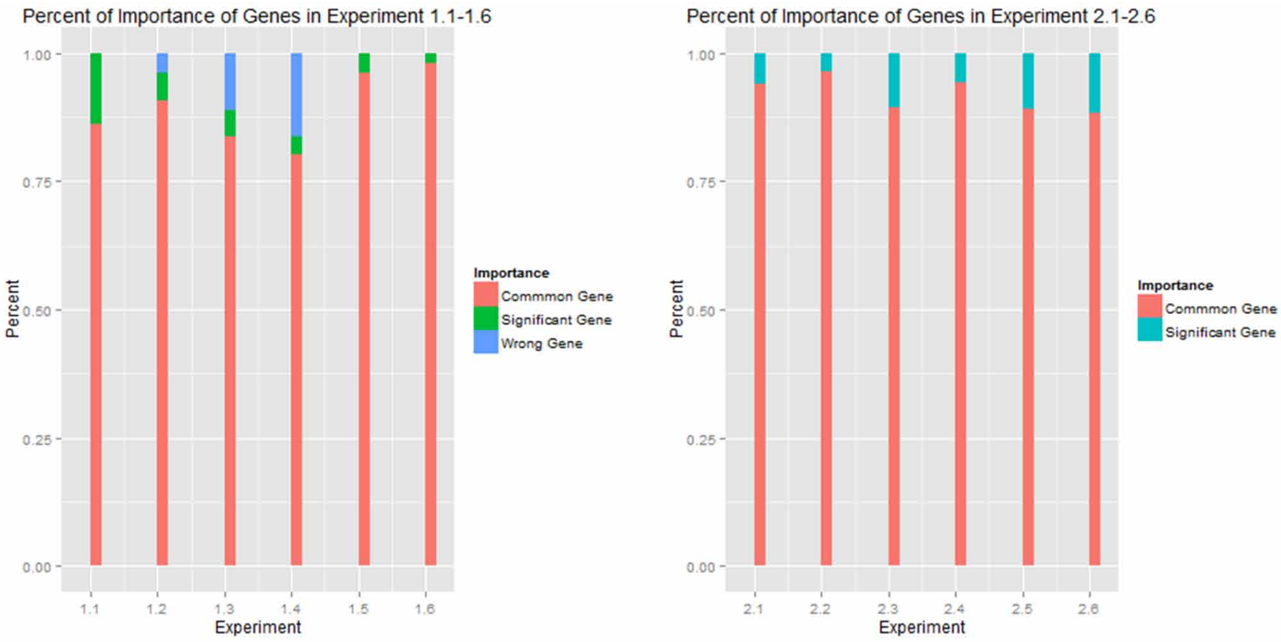

Supplementary Figure S5: Components of overlapping genes in 12 experiments, with correlation coefficient 0.9; comparing experiments 1.1 and 2.1.

Supplementary Table S1: *P*-value of rank-sum of overlapping genes (PRSOG) of experiment 1.2 with different correlation coefficients

Experiment 1.2					
Correlation Coefficient	Mean POG	RSOG	RSOG Distribution		PRSOG
			Mean	SD	
0.5	0.085	0.047	0.046	0.0005	0.11
0.6	0.085	0.047	0.046	0.0006	0.11
0.7	0.085	0.047	0.046	0.0009	0.19
0.8	0.085	0.045	0.046	0.0015	0.80
0.9	0.085	0.042	0.046	0.0026	0.95

Supplementary Table S2: *P*-value of rank-sum of overlapping genes (PRSOG) of experiment 1.3 with different correlation coefficients

Experiment 1.3					
Correlation Coefficient	Mean POG	RSOG	RSOG Distribution		PRSOG
			Mean	SD	
0.5	0.085	0.046	0.046	0.0003	0.78
0.6	0.085	0.045	0.046	0.0004	0.96
0.7	0.085	0.045	0.046	0.0005	0.94
0.8	0.085	0.045	0.046	0.0009	0.77
0.9	0.085	0.043	0.046	0.0019	0.92

Supplementary Table S3: *P*-value of rank-sum of overlapping genes (PRSOG) of experiment 1.4 with different correlation coefficients

Experiment 1.4					
Correlation Coefficient	Mean POG	RSOG	RSOG Distribution		PRSOG
			Mean	SD	
0.5	0.100	0.053	0.053	0.0003	0.52
0.6	0.100	0.052	0.053	0.0003	0.78
0.7	0.100	0.051	0.053	0.0004	0.99
0.8	0.100	0.050	0.053	0.0008	0.99
0.9	0.100	0.046	0.053	0.0017	0.99

Supplementary Table S4: *P*-value of rank-sum of overlapping genes (PRSOG) of experiment 1.5 with different correlation coefficients

Experiment 1.5					
Correlation Coefficient	Mean POG	RSOG	RSOG Distribution		PRSOG
			Mean	SD	
0.5	0.055	0.026	0.030	0.0018	0.98
0.6	0.055	0.028	0.030	0.0021	0.77
0.7	0.055	0.030	0.029	0.0027	0.47
0.8	0.055	0.032	0.029	0.0035	0.26
0.9	0.055	0.019	0.029	0.0080	0.91

Supplementary Table S5: *P*-value of rank-sum of overlapping genes (PRSOG) of experiment 1.6 with different correlation coefficients

Experiment 1.6					
Correlation Coefficient	Mean POG	RSOG	RSOG Distribution		PRSOG
			Mean	SD	
0.5	0.060	0.027	0.030	0.0020	0.94
0.6	0.060	0.025	0.030	0.0025	0.96
0.7	0.060	0.027	0.030	0.0031	0.81
0.8	0.060	0.027	0.030	0.0042	0.70
0.9	0.060	0.010	0.030	0.0101	0.97

Supplementary Table S6: *P*-value of rank-sum of overlapping genes (PRSOG) of experiment 2.2 with different correlation coefficients

Experiment 2.2					
Correlation Coefficient	Mean POG	RSOG	RSOG Distribution		PRSOG
			Mean	SD	
0.5	0.060	0.033	0.033	0.0013	0.49
0.6	0.060	0.031	0.033	0.0017	0.77
0.7	0.060	0.030	0.033	0.0022	0.84
0.8	0.060	0.031	0.033	0.0029	0.71
0.9	0.060	0.033	0.033	0.0046	0.45

Supplementary Table S7: *P*-value of rank-sum of overlapping genes (PRSOG) of experiment 2.3 with different correlation coefficients

Experiment 2.3					
Correlation Coefficient	Mean POG	RSOG	RSOG Distribution		PRSOG
			Mean	SD	
0.5	0.065	0.029	0.034	0.0017	0.99
0.6	0.065	0.030	0.034	0.0019	0.94
0.7	0.065	0.031	0.034	0.0024	0.83
0.8	0.065	0.029	0.034	0.0031	0.93
0.9	0.065	0.026	0.034	0.0043	0.95

Supplementary Table S8: *P*-value of rank-sum of overlapping genes (PRSOG) of experiment 2.4 with different correlation coefficients

Experiment 2.4					
Correlation Coefficient	Mean POG	RSOG	RSOG Distribution		PRSOG
			Mean	SD	
0.5	0.040	0.020	0.019	0.0014	0.29
0.6	0.040	0.019	0.019	0.0016	0.63
0.7	0.040	0.019	0.019	0.0020	0.58
0.8	0.040	0.019	0.019	0.0026	0.60
0.9	0.040	0.017	0.019	0.0037	0.72

Supplementary Table S9: *P*-value of rank-sum of overlapping genes (PRSOG) of experiment 2.5 with different correlation coefficients

Experiment 2.5					
Correlation Coefficient	Mean POG	RSOG	RSOG Distribution		PRSOG
			Mean	SD	
0.5	0.120	0.059	0.063	0.0023	0.98
0.6	0.120	0.055	0.063	0.0029	0.99
0.7	0.120	0.053	0.063	0.0035	0.99
0.8	0.120	0.061	0.063	0.0046	0.70
0.9	0.120	0.070	0.063	0.0074	0.18

Supplementary Table S10: *P*-value of rank-sum of overlapping genes (PRSOG) of experiment 2.6 with different correlation coefficients

Experiment 2.6					
Correlation Coefficient	Mean POG	RSOG	RSOG Distribution		PRSOG
			Mean	SD	
0.5	0.040	0.018	0.019	0.0013	0.81
0.6	0.040	0.016	0.019	0.0016	0.92
0.7	0.040	0.015	0.019	0.0020	0.95
0.8	0.040	0.017	0.019	0.0026	0.76
0.9	0.040	0.015	0.019	0.0040	0.78

Supplementary Table S11: Results of fitting power-law, log normal, exponential distribution with correlation coefficient 0.5

Experiment	Power-law distribution			Log normal distribution				Exponential distribution		
	Xmin	Parameter	KSa	Xmin	Parameter 1	Parameter 2	KSa	Xmin	Parameter	KSa
1.1	0.00097	151.5	0.084	0.00096	−6.946	0.0118	0.032*	0.00098	0.000666	0.667
1.2	0.00063	423.0	0.060	0.00062	−7.374	0.0038	0.097	0.00064	0.000619	0.750
1.3	0.00058	1019.7	0.172	0.00058	−7.484	0.0431	0.280	0.00059	0.000575	0.750
1.4	0.00062	886.0	0.180	0.00061	−7.393	0.0038	0.319	0.00064	0.000614	0.667
1.5	0.00084	151.6	0.061	0.00084	−7.078	0.0094	0.038*	0.00086	0.000576	0.667
1.6	0.00098	85.5	0.070	0.00094	−6.950	0.0233	0.033*	0.00101	0.000615	0.667
2.1	0.00100	64.9	0.099	0.00081	−7.011	0.0764	0.043*	0.00105	0.000672	0.667
2.2	0.00074	89.2	0.079	0.00072	−7.219	0.0159	0.019*	0.00076	0.000597	0.667
2.3	0.00074	165.5	0.042	0.00074	−7.207	0.0095	0.058	0.00076	0.000597	0.667
2.4	0.00076	167.3	0.023*	0.00076	−7.196	0.0139	0.043	0.00078	0.000583	0.667
2.5	0.00084	79.7	0.075	0.00077	−7.126	0.0369	0.027*	0.00087	0.000588	0.667
2.6	0.00080	90.2	0.087	0.00076	−7.162	0.0270	0.023*	0.00082	0.000559	0.667

K-S = *p*-value of Kolmogorov-Smirnov test, which is commonly used to compare a sample with a reference probability distribution or two samples;
**p*-value of K-S test has statistical significance of 0.05;
***p*-value of K-S test has statistical significance of 0.01.

Supplementary Table S12: Results of fitting power-law, log normal, exponential distribution with correlation coefficient 0.6

Experiment	Power-law distribution			Log normal distribution				Exponential distribution		
	Xmin	Parameter	KSa	Xmin	Parameter 1	Parameter 2	KSa	Xmin	Parameter	KSa
1.1	0.00141	93.9	0.056	0.00102	−6.842	0.0384	0.037*	0.00116	0.000686	0.667
1.2	0.00064	351.2	0.079	0.00063	−7.361	0.0059	0.105	0.00065	0.000620	0.667
1.3	0.00058	678.6	0.138	0.00057	−7.454	0.0042	0.221	0.00060	0.000575	0.667
1.4	0.00062	29.0	0.265	0.00062	−7.368	0.0372	0.255	0.00062	0.000615	0.667
1.5	0.00096	144.7	0.119	0.00091	−6.978	0.0220	0.042*	0.00097	0.000593	0.667
1.6	0.00115	55.2	0.070	0.00134	−6.784	0.0306	0.026*	0.00121	0.000641	0.667
2.1	0.00113	27.0	0.087	0.00106	−6.792	0.0532	0.029*	0.00126	0.000674	0.667
2.2	0.00084	126.5	0.041*	0.00077	−7.129	0.0311	0.031*	0.00086	0.000602	0.667
2.3	0.00079	123.4	0.062	0.00077	−7.144	0.0153	0.064	0.00084	0.000618	0.667
2.4	0.00084	230.4	0.040*	0.00082	−7.093	0.0116	0.032*	0.00086	0.000607	0.667
2.5	0.00095	36.9	0.078	0.00087	−6.998	0.0513	0.027*	0.00103	0.000603	0.667
2.6	0.00093	53.9	0.082	0.00086	−7.024	0.0734	0.018*	0.00097	0.000573	0.667

K-S = *p*-value of Kolmogorov-Smirnov test, which is commonly used to compare a sample with a reference probability distribution or two samples;
*p-value of K-S test has statistical significance of 0.05;
**p-value of K-S test has statistical significance of 0.01.

Supplementary Table S13: Results of fitting power-law, log normal, exponential distribution with correlation coefficient 0.8

Experiment	Power-law distribution			Log normal distribution				Exponential distribution		
	Xmin	Parameter	KSa	Xmin	Parameter 1	Parameter 2	KSa	Xmin	Parameter	KSa
1.1	0.00223	83.5	0.108	0.00174	−6.247	0.0752	0.043*	0.00224	0.000879	0.667
1.2	0.00074	186.2	0.061	0.00073	−7.229	0.0128	0.021*	0.00075	0.000630	0.667
1.3	0.00063	217.0	0.035*	0.00062	−7.385	0.0091	0.027*	0.00064	0.000579	0.667
1.4	0.00065	209.9	0.032*	0.00062	−7.352	0.0117	0.032*	0.00066	0.000617	0.667
1.5	0.00145	19.5	0.077	0.00134	−6.540	0.0711	0.031*	0.00167	0.000760	0.667
1.6	0.00209	23.5	0.054	0.00134	−6.191	0.0712	0.047*	0.00242	0.000891	0.667
2.1	0.00160	7.1	0.072	0.00120	−6.487	0.2297	0.031*	0.00252	0.000770	0.667
2.2	0.00129	24.8	0.072	0.00097	−6.794	0.1265	0.043*	0.00145	0.000649	0.667
2.3	0.00107	123.2	0.052	0.00104	−6.850	0.0165	0.049*	0.00110	0.000787	0.667
2.4	0.00118	164.0	0.084	0.00112	−6.763	0.0208	0.042*	0.00120	0.000785	0.667
2.5	0.00159	17.1	0.075	0.00104	−6.679	0.1967	0.041*	0.00189	0.000686	0.667
2.6	0.00169	31.2	0.055	0.00165	−6.403	0.0514	0.037*	0.00180	0.000674	0.667

K-S = *p*-value of Kolmogorov-Smirnov test, which is commonly used to compare a sample with a reference probability distribution or two samples;
*p-value of K-S test has statistical significance of 0.05;
**p-value of K-S test has statistical significance of 0.0.

Supplementary Table S14: Results of fitting power-law, log normal, exponential distribution with correlation coefficient 0.9

Experiment	Power-law distribution			Log normal distribution				Exponential distribution		
	Xmin	Para-meter	KSa	Xmin	Para-meter 1	Para-meter 2	KSa	Xmin	Para-meter	KSa
1.1	NAb	NA	NA	NA	NA	NA	NA	NA	NA	NA
1.2	0.00100	56.3	0.057	0.00088	−6.969	0.0530	0.032*	0.00106	0.000651	0.667
1.3	0.00080	128.3	0.060	0.00076	−7.162	0.0217	0.026*	0.00082	0.000588	0.667
1.4	0.00077	127.1	0.068	0.00075	−7.173	0.0140	0.033*	0.00079	0.000624	0.667
1.5	NA	NA	NA	NA	NA	NA	NA	NA	NA	NA
1.6	NA	NA	NA	NA	NA	NA	NA	NA	NA	NA
2.1	NA	NA	NA	NA	NA	NA	NA	NA	NA	NA
2.2	0.00239	13.2	0.088	0.00117	−6.463	0.2806	0.029*	0.00293	0.000826	0.667
2.3	0.00156	96.8	0.084	0.00148	−6.482	0.0212	0.028*	0.00160	0.000984	0.667
2.4	0.00190	65.5	0.073	0.00166	−6.330	0.0507	0.033*	0.00098	0.000984	0.085
2.5	0.00220	5.6	0.062	0.00143	−6.345	0.3726	0.027*	0.00418	0.000956	0.667
2.6	0.00246	6.9	0.088	0.00100	−6.558	0.4445	0.034*	0.00387	0.000946	0.667

K-S = *p*-value of Kolmogorov-Smirnov test, which is commonly used to compare a sample with a reference probability distribution or two samples;
* *p*-value of K-S test has statistical significance of 0.05;
** *p*-value of K-S test has statistical significance of 0.0.
